# VATS Right Upper Lobe Anterior Segmentectomy in Post Left Pneumonectomy: Technique

**DOI:** 10.3779/j.issn.1009-3419.2020.102.26

**Published:** 2020-08-20

**Authors:** Balasubramanian VENKITARAMAN, Jichen QU, Lei JIANG

**Affiliations:** 1 Department of Surgical Oncology, Thoracic Oncology Division, Sri Ramachandra Institute of Higher Education and Research, Chennai, India; 2 Department of Thoracic Surgery, Shanghai Pulmonary Disease Hospital, Tongii University School of Medicine, Shanghai 200433, China

**Keywords:** Post pneumonectomy surgery, Video-assisted thoracoscopic surgery, Resurgery in pneumonectomy, Lung cancer surgery

## Abstract

Lung resection following pneumonectomy for recurrent lung cancer is a challenging scenario. Peri-operative airway management and choice of surgical procedure are issues to be addressed by both the anesthesiologists and thoracic surgeons. We hereby report a case of anterior segmentectomy of the right upper for recurrent lung cancer, in a patient who had previously underwent pneumonectomy for primary lung cancer one year earlier. A modified conventional tracheal intubation and unique surgical techniques were applied for video-assisted thoracoscopic surgery (VATS) anterior segmentectomy of the right upper lobe in a patient with a notable mediastinal shift (following contralateral pneumonectomy), resulting in a good recovery and clinical outcome. The clinical experience is summarized in detail in this article.

## Introduction

Lung resection for recurrent lung cancer, in post pneumonectomy (of contralateral lung) pose a challenging clinical scenario, for both anesthetist and surgeon, In our knowledge, we report the first case of video assisted thoracoscopic surgery (VATS) segmentectomy with nodal dissection, following pneumonectomy, in lung cancer patient. After pneumonectomy, intrathoracic surgery is commonly performed with cardiopulmonary bypass (CPB) or extra-corporeal membrane oxygenation (ECMO) to maintain intraoperative oxygenation. However, the use of CPB or ECMO can add to the complexity and to the cost of operation as well as increase the complication rate^[1, 2]^. In our department, a modified conventional tracheal intubation and unique surgical technique was applied for this procedure.

## Clinical data and methods

A 48 years old female patient post left pneumonectomy (March 2014), was admitted to the hospital following the diagnosis of a right upper lobe nodule. Chest computed tomography (CT) revealed a ground glass opacity (GGO) of the right upper lobe lung (Fig 1A). Routine pre-operative blood tests were normal. Pulmonary function tests were: forced expiratory volume in one second (FEV_1_) 1.12 L, FEV_1_/forced vital capacity (FVC) 46.9%, vital capacity (VC) 1.57 L, and maximal voluntary ventilation (MVV) 42.11 L/min.

**1:**
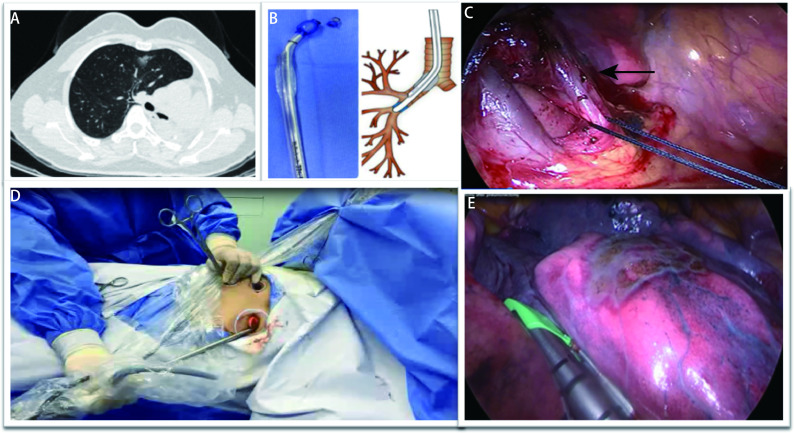
Preoperative and intraoperative figures of a patient - VATS resection of the anterior segment in the upper lobe of right lung after left pneumonectomy. A: Preoperative chest CT: Changes after left total pneumonectomy, and ground glass-like shadow in the upper lobe of right lung; B: 32-Fr left-sided double-lumen tube was used for clipping and modification to achieve a better contraposition. After anaesthesia induction, left bronchial catheter of double lumen tube was intubated into the right intermediate bronchus under the guidance of bronchoscopy to make a complete collapse in the upper lobe of right lung; C: The incision was made on both sides of the sternum; the arrow pointed to is anterior segment of right superior vein; D: A screen shot of surgical field of vision, showing the fully exposed hilum with the identification and looping of anterior segmental vein; E: Complete collapse formed in right upper lobe, with surgical staplers being applied for parenchymal transection. VATS: video-assisted thoracoscopic surgery; CT: computed tomography.

### Methods for intubation and ventilation

We had to isolate right upper lobe while ventilating middle and lower lobes to provide sufficient space for thoracoscopy. The length of intermediate bronchus was measured preoperatively with bronchoscopy and 3D reconstructions of CT images. A 32-Fr left-sided double-lumen tube was used (Fig 1B), left bronchial catheter of double lumen tube was intubated into the right intermediate bronchus under bronchoscopy guidance. Opening of the right catheter of double lumen tube was exactly opposite the orifice of upper lobe bronchus. The patient tolerated the block well and the right upper lobe was fully collapsed. The ventilation parameters were 100% fraction of inspiration O_2_ (FiO_2_), positive end expiratory pressure (PEEP) 3 cmH_2_O, I:E=1:1.5, tidal volume: 6 mL/kg, respiratory rate: 13 breaths/min-15 breaths/min. Airway pressure and end-tidal carbon dioxide (PetCO_2_) were observed closely. Blood gas analysis was normal intraoperatively and postoperatively.

### Surgical technique

First, a 3 cm incision was made at the left third intercostal space parasternally and right thoracic cavity which had herniated to left, entered. This was used to place the thoracoscope (Fig 1C).

The lesion was found located in the anterior segment of the right upper lobe and remaining lung was normal. With right middle and lower lobes being ventilated, exposure of the hilum was poor. Two additional ports were placed at the right 3^rd^ intercostal space parasternally (utility incision) and the other at 4^th^ intercostal space along mid-clavicular line (used for traction) (Fig 1C).

With the hilum exposed, superior pulmonary vein was dissected, and the two branches for the anterior segment were identified (Fig 1D). These were looped and transected using stapling device. Mediastinal pleura incised along inferior margin of azygos vein and apical branch of the pulmonary artery was identified and divided. The anterior segmental bronchus was dissected free and transected. The horizontal fissure and the inter-segmental planes were cut with staplers (Fig 1E). Lymph nodes dissection was done. The frozen-section revealed malignancy with resection margin being negative. With the use of these port positions, problems of post-pneumonectomy lung herniation and limited space due to middle and lower lobes ventilation, was effectively solved. The disadvantage of the limited exposure was overcome by use of unidirectional mode of the operation, namely, to deal with veins firstly, then arteries, bronchus and fissure finally. The operation was completed in 20 min.

The patient had uneventful postoperative stay and discharged on 6^th^ day. The final histopathology showed the right upper lobe adenocarcinoma, stage Ia (T1N0M0). At 3 years follow up, contrast enhanced CT scan of chest and a bronchoscopy was done, which were within normal limits, without any evidence of recurrent disease. On clinical assessment, patient was doing well, able to perform daily activities.

## Discussion

Patients receiving pneumonectomy may require another operation in case a new neoplasm arises in the other lung. The majority of such cases are operated with use of extra-corporeal circulation or ECMO, latter being technically easier than the former, though it is expensive and has certain complications^[2]^, making this choice unattractive

The lung function could basically reach 60%-80% of the preoperative level, 1 year postoperatively, reaching up to 50%-70% of the predicated value^[3]^. In this study, the patient had a great improvement in lung function 2 years after surgery and could tolerate lung resection.

The method described above has a few advantages, in patients undergoing thoracic surgery after pneumonectomy, like use of a conventional double lumen tube, which is simple, convenient and easy to master and cost effective and with fewer postoperative complications compared to CPB or ECMO.

## Conclusion

Owing to complexity of limited exposure, such cases should be performed at specialized centers by experienced surgeons. In our opinion lung resections in patients who have been previously offered a pneumonectomy, pose a major challenge for both the surgeon and the anesthesiologist.

## Acknowledgment

A great number of people had gone to considerable lengths to help this work, among whom we would single out here the name of Prof Kostis Marios Soultanis, Jiaan Ding, and Chang Chen. We thank Guangyu Chen for the illustration and statistical analyses. For all their kind help and hard work, we wish to express our heartfelt gratitude.

## Author contributions

Venkitaraman B, Jiang L and Qu JC participated in the design of the study and performed the statistical analysis, participated in its design and coordination, and helped to draft the manuscript. All authors read and approved the final manuscript.
